# Low caregiver state anxiety is associated with worse glycemic control in youth with type 1 diabetes mellitus: a cross-sectional study

**DOI:** 10.3389/fped.2026.1806430

**Published:** 2026-06-24

**Authors:** Camilia Kamoun, Jane C. Khoury, Wael Shamseddeen, Mohamad El Zein, Nancy Abigail Crimmins, Pascale Nawfal, Hala Mounir Tfayli

**Affiliations:** 1 Department of Pediatrics, Cincinnati Children's Hospital Medical Center (CCHMC), Cincinnati, OH, United States; 2 Department of Pediatrics, Division of Pediatric Endocrinology, University of North Carolina at Chapel Hill School of Medicine, Chapel Hill, NC, United States; 3 Division of Endocrinology, CCHMC, Cincinnati, OH, United States; 4 Division of Biostatistics and Epidemiology, CCHMC, Cincinnati, OH, United States; 5 Department of Environmental and Public Health Sciences, Division of Epidemiology, University of Cincinnati, Cincinnati, OH, United States; 6 Department of Pediatrics, College of Medicine, University of Cincinnati, Cincinnati, OH, United States; 7 Department of Psychiatry, Faculty of Medicine, American University of Beirut, Beirut, Lebanon; 8 Department of Pediatrics and Adolescent Medicine, Division of Endocrinology, Faculty of Medicine, American University of Beirut, Beirut, Lebanon; 9 Chronic Care Center, Diabetes Unit, Beirut, Lebanon

**Keywords:** adolescents, caregiver anxiety, glycemic control, psychological factors, type 1 diabetes mellitus, pediatric

## Abstract

**Introduction:**

The impact of caregiver anxiety on management of type 1 diabetes (T1D) is not well understood. This study examined the association between caregiver anxiety, glycemic control, and anxiety symptoms in youth with T1D.

**Methods:**

We conducted a cross-sectional study of 200 dyads of youth aged 11–17 years, with T1D duration >1 year and their primary caregivers. Caregiver anxiety was assessed by the State-Trait Anxiety Inventory (STAI), and youth anxiety by the Screen for Child Anxiety Related Emotional Disorders (SCARED). Multiple logistic regression examined associations between STAI quartiles and glycemic control defined as HbA1c ≤ 7.5%.

**Results:**

Youth with caregivers in the lowest quartile of state anxiety had lower odds of HbA1c ≤ 7.5% compared to the middle 50% [adjusted odds ratio = 0.35; 95% CI (0.14–0.84), *p* = 0.018]. Possible anxiety disorder based on SCARED scores was identified in 60.5% of youth. Higher caregiver STAI-scores correlated with higher youth SCARED-scores.

**Conclusions:**

Low caregiver state anxiety was associated with worse glycemic control in youth with T1D, a counterintuitive finding that may reflect differences in caregiver involvement. Anxiety symptoms were highly prevalent among youth in this cohort. These findings highlight the complex interplay between caregiver anxiety, youth anxiety and glycemic control, and support the importance of psychological screening, and family-centered approaches in routine diabetes care.

## 1 Introduction

Caregiver psychological distress, including anxiety, has been associated with impaired diabetes management, higher HbA1c levels, and worse quality of life among youth with type 1 diabetes mellitus (T1D) (1–6). Caregivers of youth with T1D are reported to have higher prevalence of anxiety symptoms compared to caregivers of youth without chronic disease (6, 7) and these symptoms seem to be more pronounced among those whose children have suboptimal glucose control (6). Parental anxiety has been shown to influence the development of youth anxiety (8), and existing evidence links youth anxiety with poor glycemic control, reduced diabetes self-management, and impaired diabetes-specific quality of life (9, 10). Nevertheless, the relationship between caregiver anxiety and outcomes such as glycemic control and youth anxiety remains insufficiently understood, particularly outside of North America and Europe.

Lebanon is a middle-income country situated in a region with relatively higher mental health burden compared to the global average (11). Data on psychological factors affecting youth with T1D in this context are limited. To our knowledge, no published studies from the region have examined caregiver anxiety in relation to glycemic control and anxiety among youth with T1D.

The State-Trait Anxiety Inventory (STAI) is one of the commonly used tools for assessing anxiety in caregivers in the context of T1D (12, 13) and has been validated for Lebanese adults (14). The STAI distinguishes between two components: situational anxiety (State) and anxiety as a more enduring characteristic (Trait) (15). While some studies have examined caregiver trait anxiety (Cameron, 2007) and others reported on state anxiety (6), or both (13), the differential effects of caregiver state vs. trait anxiety in youth with T1D remains poorly understood. Moreover, to our knowledge, no prior studies have specifically examined the association of low caregiver anxiety and glycemic control in T1D.

The primary aim of this study was to assess state and trait anxiety among caregivers of youth with T1D in Lebanon, and to examine its association with glycemic control and youth anxiety symptoms. Based on clinical observation, we hypothesized a non-linear relationship whereby both very low and very high levels of state and trait caregiver anxiety would be associated with worse glycemic control, compared to moderate anxiety levels. We also hypothesized a positive correlation between caregiver and youth anxiety symptoms (8). A secondary *post-hoc* aim was to assess the prevalence of anxiety symptoms among youth with T1D in this cohort.

## 2 Methods

A cross-sectional study of dyads of youth with T1D and their primary caregivers was conducted at two sites in Beirut, Lebanon: the pediatric endocrinology clinic at the American University of Beirut Medical Center (AUBMC) and the pediatric diabetes clinic at the Chronic Care Center (CCC). Participants were recruited during routine clinic visits between January and July 2019. The study was approved by the Institutional Review Boards of the participating institutions (AUBMC: *protocol approval number SBS-2018-017,* Cincinnati Children's Hospital Medical Center: *protocol approval number 2018-4537*). Written informed consent/assent was obtained from all participants. Participants who declined full participation but met eligibility criteria, were invited to provide written informed consent/assent to collection of basic demographic information.

### 2.1 Participants inclusion and exclusion criteria

Youth 11 to 17 years old, with a diagnosis of T1D ≥1 year, who were accompanied by their primary caregiver on the day of the visit were eligible for inclusion. The age range was chosen based on the validated Arabic versions of the study tools. A primary caregiver was defined as the individual responsible for more than 50% of the child's diabetes care and had to be older than 18 years. In instances of equally shared caregiving, the caregivers selected one individual to participate. All participants were required to be able to read basic Arabic or English and to complete self-administered anxiety assessment measures independently.

Exclusion criteria included: profound to moderate developmental disability in the youth, an established psychiatric disorder in either the youth or the caregiver, or the presence of serious unrelated medical conditions. Patients with well-managed co-morbid diagnoses (e.g., thyroid disease) were not excluded. Patients and caregivers with pre-diagnosed and/or treated psychiatric conditions, including anxiety, were excluded.

### 2.2 Measurement of participants’ anxiety symptoms

Caregiver anxiety using the State-Trait Anxiety Inventory (STAI; rights for use purchased from Mind Garden Inc.) (15). The STAI consists of 40 items scored on a 4-point Likert scale: the first 20 items measure state anxiety (which reflects emotional responses at a specific point in time, and the second 20 items assess trait anxiety (which captures anxiety as a long-standing personality trait) (15). Each subscale yields a score ranging from 20 to 80, with higher scores indicating greater anxiety (15). The Arabic version of the STAI used was the official translation provided by Mind Garden Inc. The STAI was administered according to standard instructions: state items were completed based on “how you feel right now, at this moment” and trait items based on “how you generally feel.” At the time of consent, participants were informed that the study focused on anxiety in the context of type 1 diabetes, but no diabetes-specific wording were applied to the STAI items.

Youth anxiety symptoms were measured using both the child and parent-proxy versions of the Screen for Child Anxiety Related Emotional Disorders (SCARED) (16). The SCARED, both youth and parent-proxy versions, consist of 41 items scored on a 3-point scale. A SCARED score of ≥25 (out of 82) is concerning for possible anxiety, while scores >30 are more specific for true presence of an anxiety disorder (16, 17).

These tools were chosen because they are validated across diverse populations (14, 18–20) and specifically validated for the Lebanese population (14, 19). (See Supplementary Methods for details). In our sample Cronbach's alpha coefficients were as follows: 0.84 for STAI-State, 0.92 for STAI-Trait, 0.90 for youth SCARED, and 0.93 for caregiver SCARED, indicating good to excellent internal consistency. Youth and caregivers completed their SCARED and STAI assessments independently with their preferred language (Arabic or English). Participants completed the measures in a calm room at AUBMC or CCC clinics.

### 2.3 Clinical and demographic data collection

A structured questionnaire, developed for this study (Supplementary Material S1), was administered by a trained research assistant. Adherence and glycemic control data were collected through both self-report and glucose monitoring devices for the month preceding the study visit. Data from the first and second two weeks of the month were compared to assess for a potential “white coat” effect on glycemic control (21). Severe hypoglycemia was defined as any episode involving seizure or loss of consciousness. Hypoglycemia was defined as any glucose value below 70 mg/dL (3.9 mmol/L). HbA1c values were measured using ion-exchange high-performance liquid chromatography (The Bio-Rad Variant II HbA2/HbA1c Dual Program, Bio-Rad Laboratories, Inc. CA, USA). All study surveys were administered on an electronic tablet using RedCap® (Research Electronic Data Capture), a secure, web-based application designed to support data capture for research studies, hosted at Cincinnati Children's Hospital Medical Center (22).

### 2.4 Statistical analysis

Study population size was determined based on an estimate that a sample size of 200 would be necessary for 80% power, allowing for a type 1 error of 0.05 and a 2-sided test to detect a 50% lower odds of HbA1c out of target range [>7.5% (58.5 mmol/mol)] in the study participants for a caregiver within the middle 50% of the distribution of anxiety scores allowing for potential co-variates in the model. This sample size also allowed detection of a correlation of r = 0.2 between caregiver and youth anxiety, with 80% power and an alpha of 0.05 (two-tailed), allowing for up to six additional covariates in a regression model (SAS, version 9.4, SAS Institute Inc., Cary, NC, USA; PROC POWER). Statistical analyses were conducted using SAS® version 9.4 (SAS Institute Inc., Cary, NC, USA) and Stata/MP© version 16.1 (StataCorp, College Station, Texas, USA). A *p*-value of <0.05 was considered statistically significant.

Continuous variables were checked for distributional properties and outliers, and categorical variables were checked for valid coding. Continuous data are reported as mean (standard deviation) or median (25th, 75th percentiles; IQR) plus range, as appropriate; categorical variables are reported as frequency (percentage). Agreement between blood glucose monitoring devices and self-reported glucose control was assessed using the kappa or weighted kappa statistic, as appropriate. No significant difference was observed between glycemic data from the first vs. the second two-week period of the month, thus data from the entire month were used in subsequent analyses. Difference between groups for continuous variables was assessed using the t-test or Wilcoxon Rank Sum, as appropriate, and for categorical variables using Chi-square tests.

The distribution of STAI state and STAI trait in our sample deviated from the normality assumption, and the relationship between caregiver anxiety and glycemic control has been reported to be non-linear in previous studies (12, 23, 24) Accordingly, as planned *a priori*, the STAI scores were partitioned at the 25th and 75th percentiles and examined as the independent variables of interest for the association with HbA1c as the dependent variable, using ≤7.5% (58.5 mmol/mol) as the pre-defined cut-point. This cut-point was the recommended target HbA1c at the time of study design. The primary study hypothesis, based on repeated clinical observation at our sites, that both very low and very high caregiver state anxiety might be associated with worse glycemic control motivated testing the middle 50% as the reference group (moderate anxiety). Multiple logistic regression was used to examine the association between caregiver anxiety levels (STAI-state and STAI-trait) and glycemic control (HbA1c ≤ 7.5%). Initial models included variables chosen by examination of the associations with the dichotomized HbA1c or categorized independent variable of interest (STAI state or trait). The specific variables included in the initial model are listed in a footnote of the tables showing the results for the analysis of the independent variable of interest. Backward elimination was used to select the final model with careful examination of the effect on the independent variables of interest at each step. Specifically, the covariates and confounders were selected using a *p*-value cut-point of <0.20 for inclusion in the initial model and then backward elimination was used for inclusion in the final model. The most non-significant being removed first and the effect on the variable of interest inspected; if the latter effect was greater than 10% the variable was returned to the model. Multiple regression with STAI state out to third degree and a restricted cubic spline was also used to further examine the association of STAI state as continuous variables with HbA1c as continuous variables. However, it was decided that the initial approach was the most clinically meaningful, and is reported as the primary result. Additional analyses treating STAI-state as an interval measure and HbA1c as both categorical and interval measure, and treating STAI-state as quartiles and HbA1c as an interval measure were performed and are included in the Supplementary Material. (These additional analyses were not done with STAI trait due to the lack of association between STAI trait and HbA1c in the initial approach for both bivariate and multivariable analyses.)

Pre-planned subgroup analyses were conducted by youth age group (11–13 years vs >13–17 years), based on evidence that caregiver anxiety may differentially affect younger vs. older adolescents (12). Similarly, sex-stratified subgroup analyses were conducted. The subgroup analyses were not included in the original power considerations. Pearson correlation was used to examine the relationship between caregiver STAI scores and youth anxiety as measured by SCARED scores.

## 3 Results

### 3.1 Study population characteristics

A total of 234 potential caregiver-youth dyads were approached, of whom 200 (85%) consented to participate. Among those who declined, 21 (62%) consented to collect demographic information. No significant differences in age, sex, diabetes duration or HbA1c were found between consenters and non-consenters (Supplementary Table 1). The study group included three sibling pairs; one caregiver completed the survey for both siblings. Sixteen dyads completed the study measures in English, while the remainder completed them in Arabic. The characteristics of the youth and caregiver participants are summarized in Table 1.

**Table 1:** Study group characteristics; overall and by HbA1c categorization.

Youth Characteristics *n* = 200	Overall	HbA1c ≤7.5% *N* = 65	HbA1c >7.5% *N* = 135	*p*-value
Age, years	14.1 ± 1.7 [11.0–17.0]	13.8 ± 1.8 [11.0–17.0]	14.2 ± 1.7 [11.0–17.0]	0.103
Age 11–13, years	59 (29.5)	24 (36.9)	35 (25.9)	0.110
Female	111 (55.5)	37 (56.9)	74 (54.8)	0.779
Diabetes duration, years	5.8 ± 3.2 [1.0–15.3]	5.5 ± 3.7 [1.0–15.3]	6.0 ± 3.0 [1.0–14.3]	0.316
Body Mass Index (kg/m^2^)	22.3 ± 3.89 [14.2–38.8]	21.8 ± 3.2 [16.0–29.8]	22.5 ± 4.2 [14.2–38.8]	0.297
Co-morbid conditions				0.840 (for any comorbid condition)
Thyroid	11 (5.5)	5 (7.7)	6 (4.4)	
Celiac	5 (2.5)	2 (4.6)	2 (1.5)	
Seizure	3 (1.5)	0	3 (2.2)	
Other	6 (3.0)	0	6 (4.4)	
Any	26 (13.0)	8 (12.3)	18 (13.3)	
HbA1c (%)	8.5 ± 1.6 [5.2–15.0]	7.0 ± 0.5 [5.2–7.5]	9.2 ± 1.4 [7.6–15.0]	<0.0001
Insulin regimen				0.268
Pump	27 (13.5)	12 (18.5)	15 (11.1)	
Injections: Basal-bolus or Combination long + short acting	173 (86.5)	53 (81.5)	120 (88.9)	
CGM or flash monitoring used	52 (26.0)	24 (36.9)	28 (20.7)	0.001
Severe hypoglycemia^†^ ever	54 (27.0)	17 (26.2)	37 (27.4)	0.852
DKA after diagnosis	15 (7.5)	3 (4.6)	12 (8.9)	0.394
Hypoglycemia frequency^^^ youth	3.5 (3, 4) [1, 5]	4.0 (3, 4) [1–5]	3.0 (2, 4) [1–5]	0.008^^^^
Hypoglycemia frequency^^^ caregiver	3.0 (2, 4) [1, 5]	4.0 (3, 4) [1–5]	3.0 (2, 4) [1–5]	0.003^^^^
Severe hypoglycemia^†^ in past 3 months	4 (2.0)	2 (3.1)	2 (1.5)	0.597
Who gives insulin				0.642
Caregiver only	7 (3.5)	1 (1.5)	6 (4.4)	
Youth only	144 (72.0)	47 (72.3)	97 (71.8)	
Both	49 (24.5)	17 (26.2)	32 (23.7)	
Who determines insulin amount				0.720*
Caregiver only	27 (13.5)	8 (12.3)	19 (14.1)	
Youth only	90 (45.0)	27 (41.5)	63 (46.7)	
Both	82 (41.0)	29 (44.6)	53 (39.3)	
N/A (fixed doses)	1 (0.5)	1 (1.5)	0	
Who counts carbohydrates				0.493*
Caregiver only	22 (11.0)	6 (9.2)	16 (11.8)	
Youth only	72 (36.0)	23 (35.4)	49 (36.3)	
Both	85 (42.5)	33 (50.8)	52 (38.5)	
N/A	21 (10.5)	3 (4.6)	18 (13.3)	

Caregiver characteristics *n* = 197	Overall	HbA1c ≤7.5% *N* = 65	HbA1c >7.5% *N* = 135	*p*-value
Age, yrs	43.3 ± 7.5 [18.0, 70.0]	44.2 ± 7.1 [30.0–70.0]	42.8 ± 7.7 [18.0–63.0]	0.220
Females	162 (82.2)	55 (84.6)	109 (80.7)	0.504
Relationship to patient				
Mother	158 (80.2)	54 (83.1)	106 (78.5)	0.572 mother vs. others
Father	34 (17.3)	10 (15.4)	25 (18.5)	
Grandmother	1 (0.5)	1 (1.5)	0	
Sibling	4 (2.0)	0	4 (3.0)	
Family structure				
Single-family	179 (90.9)	62 (95.4)	119 (88.1)	0.126 single vs. others
Multi-family	3 (1.5)	0	4 (3.0)	
Multi-generational	15 (7.6)	3 (4.6)	12 (8.9)	
Marital status				
Married	179 (90.9)	61 (93.8)	121 (89.6)	0.433 married vs. others
Divorced	10 (5.1)	3 (4.6)	7 (5.2)	
Widowed father	2 (1.0)	0	2 (1.5)	
Widowed mother	5 (2.5)	1 (1.5)	4 (3.0)	
Unmarried, parents living together	0 (0.0)	0	0	
Unmarried, parents living separately	1 (0.5)	0	1 (0.7)	
Education level				
Primary	71 (36.0)	18 (27.7)	54 (40.0)	0.188
Secondary	45 (22.8)	14 (21.5)	32 (23.7)	
Technical degree	20 (10.2)	7 (10.8)	14 (10.4)	
University degree	61 (31.0)	26 (40.0)	35 (25.9)	
Caregiver employed	86 (43.6)	26 (40.0)	61 (45.2)	0.488
Caregiver diabetes				0.278 no diabetes vs. any diabetes
T1D	5 (2.5)	0	5 (3.7)	
Insulin dependent T2D	2 (1.0)	1 (1.5)	1 (0.7)	
Non-insulin dependent T2D	10 (5.1)	2 (3.1	8 (5.9)	
No diabetes	180 (91.4)	62 (95.4)	121 (89.6)	
Family income^‡^				0.129
<$450	15 (7.6)	2 (3.1)	13 (9.6)	
$450-$800	46 (23.4)	13 (20.0)	33 (24.4)	
$801-$1,500	91 (46.2)	30 (46.2)	64 (47.4)	
$1,501-$4,000	37 (18.8)	15 (23.1)	22 (16.3)	
>$4,000	8 (4.1)	5 (7.7)	3 (2.2)	
Trouble paying for medications and supplies	89 (45.2)	26 (40.0)	63 (46.7)	0.374
Trouble obtaining medications and supplies	35 (17.8)	10 (15.4)	25 (18.5)	0.585
Caregiver does not receive help from other caregivers for DM management	132 (67.0)	42 (64.6)	93 (68.9)	0.546

Continuous variables presented as mean ± SD [range], or median (25th, 75th percentile) [range]. Categorical variables presented as *n* (%).

*p*-value assessed by T-test, Wilcoxon rank sum, Chi-square or Fisher's exact test, as appropriate.

† Severe hypoglycemia defined as seizure or loss of consciousness from hypoglycemia.

^ 1 = none, 2 = very few <3x/mo, 3 = few 4–8x/mo, 4 = frequent 9–15/mo, 5 = almost daily, 6 = unable to assess, 7 = n/a.

^^ Unable to assess and N/A excluded.

* N/A excluded.

‡ Data collected before the current Lebanese economic crisis and Lebanese Lira devaluation (by greater than 95% its value in late 2019).

HbA1c was ≤7.5% (58.5 mmol/mol) among 32.5% (*n* = 65) of youth. These youth were more likely than those with HbA1c > 7.5% (58.5 mmol/mol) to have a higher family income (*p* = 0.012, test for trend), to use a flash glucose monitoring system or continuous glucose monitor (37% vs 21%, respectively; *p* = 0.01), and more frequent hypoglycemia based on recall (*p* = 0.008). No other differences in demographics or clinical characteristics were found between the two groups (Table 1).

### 3.2 Caregiver anxiety measures (STAI-state and STAI-trait)

Caregiver STAI results are summarized in Table 2. The median STAI-state score of the caregivers was 32.5, IQR (25, 42.5) and their median STAI-trait score was 40.0, IQR (34.0, 46.0).

**Table 2:** Results of anxiety measures.

**Caregiver Anxiety—STAI**	Median (IQR) [range]
State	32.5 (25.0, 42.5) [20.0–72.0]
Trait	40.0 (34.0, 46.0) [23.0–74.0]

**Youth Anxiety—SCARED**	Median (IQR) [range]
Youth-reported	24.5 (17.0–34.0) [2.0–61.0]
Caregiver-reported	18.0 (10.5–30.5) [0.0–70.0]

**SCARED scores positive for anxiety, % (n)**	**≥25**	**>30** ^a^
Youth-reported SCARED only	21.0 (42)	18.5 (37)
Caregiver-reported SCARED only	10.5 (21)	8.0 (16)
Both Youth-reported SCARED and Caregiver-reported SCARED	29.0 (58)	17.0 (34)
Total (Youth- and/or Caregiver-reported)	60.5 (121)	43.5 (87)

a score that is considered more specific.

In simple bivariate analysis, higher STAI-trait scores were associated with lower caregiver education (*p* = 0.005), lower family income (*p* = 0.006), and increased self-reported hypoglycemia frequency (youth-recall *p* = 0.043; caregiver-recall *p* = 0.011). STAI-trait scores were not significantly associated with device-reported hypoglycemia frequency (*p* = 0.11). For STAI-state scores no variables except HbA1c were significantly related at *p* < 0.05.

### 3.3 Association of caregiver anxiety measures and youth glycemic control

In the regression model, youth whose caregivers had STAI-state scores in the bottom quartile had lower odds of achieving a HbA1c ≤ 7.5% (58.5 mmol/mol) [adjusted OR 0.35, 95% CI (0.14–0.84), *p* = 0.018], compared to those with scores in the middle 50% (Table 3). Details of the model are included in Table 3 footnote; adjustments were made for caregiver-reported frequency of hypoglycemia and living in a single-family home [both of which were associated with higher likelihood of HbA1c ≤ 7.5%] (Table 3). Using objective hypoglycemia data according to glucometer or flash glucose monitor (which was available only for 188 participants) yielded a slightly lower aOR of 0.30 [95% CI: (0.12–0.76), *p* = 0.011]. High STAI-state score (in the top quartile) was not associated with decreased odds of HbA1c ≤ 7.5% compared to the middle 50%. Similar results were found when the analysis was performed using HbA1c as an interval variable, (Supplementary Table 2).

**Table 3:** Association between caregiver STAI state and HbA1c.

	All youth	Ages 11–13y (*n* = 59)	Ages >13–17y (*n* = 141)
Caregiver STAI state score	aOR (95% CI) for HbA1c ≤ 7.5%*	aOR (95% CI) for HbA1c ≤ 7.5%	aOR (95% CI) for HbA1c ≤ 7.5%
**Bottom quartile** (score 20–24)	0.35 (0.14, 0.84) ***p*** **=** **0.018**	0.61 (0.13, 2.82) *p* = 0.52	0.28 (0.09, 0.81) ***p*** ***=*** **0.02**
**Middle 50%** (score 25–42)	**reference**	**reference**	**reference**
**Top quartile** (score 43–72)	0.79 (0.38, 1.64) *p* = 0.53	2.86 (0.76, 10.70) *p* = 0.12	0.43 (0.17, 1.11) *p* *=* 0.08
**Frequency of hypoglycemia—caregiver**	1.44 (1.09, 1.89) ***p*** **=** **0.01**	1.65 (0.97, 2.82) *p* = 0.06	1.51 (1.05, 2.15) ***p*** **=** **0.03**
**Living in a single-family home (yes)**	3.20 (0.87, 1.77) *p* = 0.08	Not applicable	Not applicable
	Overall *p* = 0.06 R-square = 0.12	Overall *p* = 0.18 R-square = 0.13	Overall ***p*** **=** **0.03** R-square = 0.12

*HbA1c ≤ 7.5% (58.5 mmol/mol).

The model including all youth was adjusted for frequency of hypoglycemia over the last month as reported by the caregiver and living in a single-family home.

The models by age groups were adjusted for frequency of hypoglycemia over the last month as reported by the caregiver only.

Initial models for the (All youth) and the (Age group>13–17) included: STAI state categories, age (11–13, >13–17 – overall model only), duration of diabetes, body mass index of youth, any comorbid condition as reported by the caregiver (yes vs no), use of flash glucose monitor or CGM (yes vs no), frequency of hypoglycemia as reported by the caregiver (where:1 = none, 2 = very few <3x/month, 3 = few 4–8x/month, 4 = frequent 9–15/month, 5 = almost daily), highest educational level of caregiver (primary, secondary, technical degree, university degree), family income (1 = <$450, 2 = $450-$800, 3 = $801-$1,500, 4 = $1,501-$4,000, 5 = >$4,000) family structure – single family home (yes vs no), trouble paying for medications (yes vs no).

Backward elimination was used to obtain the final model, with the most non-significant covariate being eliminated first from the model and the effect on the independent variable of interest was inspected, if the latter effect was a greater than a 10% change the variable was returned to the model.

Although the testing of the groups ages 11–13 and >13–17 had been preplanned we note that the *p*-value for the interaction of categorical STAI state and categorical age was 0.09. Due to the relatively small sample size in the 11–13 age group, we decided to use the same covariate in the above reported analysis as used for the >13–17 age group to allow visual comparison of the results for categorized STAI state.

No simple linear association was observed between HbA1c and STAI-state scores (Supplementary Table 3). However, additional analysis treating STAI-state as an interval measure and accounting for non-linear effects, revealed a significant, non-linear association between caregiver state anxiety and youth HbA1c, (*p* ≤ 0.001). The model, which incorporated significant linear, quadratic, and cubic terms for the STAI state score (*p* ≤ 0.022), explained 11%–12% of the variance in patient glycemic control, after adjusting for relevant confounders (Supplementary Table 3).

No association was found between STAI-trait score partitioned by 25th and 75th percentiles and HbA1c or in multivariable analyses.

In age-stratified subgroup analyses, the association between lower caregiver STAI-state scores and worse HbA1c was significant only among adolescents (ages >13–17 years), not pre-adolescent youth (ages 11–13 years) (Table 3 and Supplementary Table 2). However, the younger age subgroup was small (*n* = 59), limiting power to detect differences. No sex differences were detected. STAI-trait scores were not associated with differences in HbA1c sub-analysis by age and sex.

### 3.4 Youth anxiety measures (SCARED), youth-reported and caregiver-reported

SCARED scores indicated possible anxiety disorder in 60.5% of the youth, 30% by youth-self report and 30.5% by caregiver report (score >25). Using the more specific cutoff (score >30), 35.5% of the youth self-reports and 25% of caregiver-reports were positive for concern for an anxiety disorder. A detailed frequency distribution of SCARED scores is shown in Table 2.

Youth self-reported SCARED scores were significantly higher than caregiver-reported SCARED scores (mean difference of 4.6 ± 13.0 points; range: −34 to 39 points; *p* < 0.0001). Interclass correlation (ICC) between youth-reported and caregiver-reported SCARED scores demonstrated weak agreement (ICC = 0.52). Higher caregiver-reported SCARED scores were associated with lower caregiver education (*p* < 0.0001) and lower family income (*p* = 0.003). Youth-reported SCARED scores were not associated with any of the clinical or demographic variables. Mean caregiver- and youth-reported SCARED scores were lower for youth with HbA1c ≤ 7.5% (58.5 mmol/mol), compared to those with HbA1c > 7.5% (58.5 mmol/mol) (18.9 ± 12.8 vs 23.3 ± 14.8, *p* = 0.044; 23.8 ± 12.6 vs 27.7 ± 12.9, *p* = 0.045 for caregiver- and youth-reported scores respectively).

### 3.5 Correlation between caregiver and youth anxiety

Higher caregiver STAI trait and state anxiety scores correlated with higher youth SCARED anxiety scores, both by self-report and caregiver-report. Correlations were stronger between caregiver STAI and caregiver-reported SCARED scores than with youth self-reported scores (Table 4).

**Table 4:** Correlation between youth and careviger anxiety.

	STAI State	STAI Trait
Pearson correlation coefficient (*p*-value)		
SCARED Youth-reported	0.14 (**0.04**)	0.22 (**0.001**)
SCARED Caregiver-reported	0.30 (**<0.0001**)	0.46 (**<0.0001**)

## 4 Discussion

In this study, low caregiver state anxiety was associated with worse glycemic control in youth with T1D. In addition, a notably high proportion of youth screened positive for possible anxiety disorder. Caregiver anxiety correlated with youth anxiety, with stronger associations observed for caregiver-reported youth symptoms.

The observed association between lower caregiver state anxiety and reduced odds of youth achieving target HbA1c is a novel finding. One possible explanation is that very low state-anxiety scores may reflect decreased diabetes-related engagement, which mediates worse glycemic control compared to caregivers in the middle or top quartiles (25). The absence of a similar association for caregiver trait anxiety supports this hypothesis, as trait anxiety reflects an enduring psychological predisposition rather than a context-dependent state. In contrast, state anxiety assessed in the clinical setting may capture situational, diabetes-related concern more directly linked to caregiver engagement and disease management behaviors. Parenting style may also moderate this relationship; prior research suggests that collaborative parenting and increased parental autonomy-support are associated with better treatment adherence and glycemic outcomes, with effects differing by adolescent age (23, 24). To our knowledge, no prior studies examined the association between low caregiver state or trait anxiety and glycemic control.

The lack of association between high caregiver state anxiety and poor glycemic control aligns with prior studies (23, 26, 27). Previous findings on trait anxiety are mixed. While no association was found between parental trait anxiety and glycemic control in children aged 2–6 years (13), higher caregiver trait anxiety was linked to elevated HbA1c in adolescents aged 13–18 years in New Zealand (12) and the U.S (28, 29). In one study, this effect was age-specific, with higher maternal trait anxiety associated with higher HbA1c only in younger adolescents (12). Unlike these studies, we found no association between caregiver trait anxiety and glycemic control. This discrepancy may be partly explained by cultural differences in parenting styles, including varying levels of youth autonomy, which may mediate this relationship (23). Additional factors such as differences in study design, the tools used to assess anxiety, and the settings in which questionnaires were administered may also contribute. Furthermore, the exclusion of caregivers with known anxiety disorders in our study may have influenced this finding; however, the number of participants excluded for this reason was small (<5).

The global prevalence of anxiety among youth is reported to be 6.5% (30), while in Lebanon 22.6% of youth ages 8–17 years old from the general population screened positive for possible anxiety disorder (31). Among youth with T1D, anxiety symptoms are reported in 13–21.3% (9). In our cohort, 60.5% of youth screened positive for possible anxiety disorder, substantially higher than reported values in general and youth with T1D. Consistent with prior studies, caregiver anxiety scores correlated with youth anxiety as measured by SCARED (8), and youth-reported SCARED scores exceeded caregiver-reported SCARED scores (32, 33), reflecting differential detection of anxiety features between parents and children (32, 33).

The clinical implications of our findings are threefold. First, very low caregiver state anxiety may serve as a clinical signal for risk of suboptimal glycemic control. Second, our results support the role of the assessment of both caregiver and youth anxiety in optimizing glycemic control. While lower state anxiety in caregivers was associated with worse glycemic control, youth with lower SCARED scores had lower HbA1c in our study. These findings suggest that there may be distinct effects of caregiver vs. youth anxiety and trait vs. state anxiety on glycemic control. Finally, the high prevalence of youth anxiety in this study raises concern for unaddressed mental health needs that may be impacting diabetes-related health. Anxiety in youth has been associated with suboptimal glycemic control, decreased frequency of blood glucose monitoring (34–36), and increased illness-related parenting stress (37), which in turn affects glycemic control (3).

Strengths of the study include a relatively large sample size, and the assessment of both trait and state caregiver anxiety. Investigation of both forms of anxiety was important given the discrepancies in prior studies that looked at either state or trait caregiver anxiety (12, 13, 23, 27, 28). Well-described limitations for collection of adherence and glycemic control related data include potential overestimation of adherence and inaccuracy from self-report, and device malfunction and problems with data download with glucose monitors (38). Recognizing this, data was collected through both self-report and blood glucose monitors during the month preceding the study visit, improving the quality of the data.

Limitations of the study include its cross-sectional design, which precludes assessment of temporal relationships between caregiver anxiety and glycemic control; therefore, findings should be interpreted as associations rather than causal effects. Bias in response to anxiety measures is also possible; as caregivers and youth may have influenced each other's answers. This was mitigated by having them complete the anxiety measures separately. Another limitation is the assessment of caregiver anxiety symptoms rather than formal clinical diagnoses. It is possible that use of STAI scores in the upper distribution of scores was not a sensitive enough measure of significant caregiver anxiety that affects youth glycemic control, compared to clinical evaluation for anxiety. Unfortunately, diagnostic interviews were outside the scope of what was possible in the study setting. In addition, diabetes-related quality of life, including diabetes related worry or fear of hypoglycemia, factors known to be associated with glycemic control (39) were not assessed due to clinical time constraints that made such additional data collection too burdensome. Similarly, child state anxiety around diabetes was not measured, as the SCARED tool measures trait anxiety. Finally, the differential impact of child vs. caregiver anxiety on glycemic control was not examined and represents an additional limitation.

The lack of association between high caregiver anxiety and glycemic control should be interpreted with caution and within the context of our study limitations. Caregiver anxiety is an important and often underrecognized factor associated with adverse health outcomes in children with chronic diseases, particularly impacting quality of life and healthcare utilization (40, 41). Our findings should not be interpreted as suggesting a protective effect of high state caregiver anxiety on control; rather, they raise the possibility that very low caregiver state anxiety may reflect parental disengagement, placing youth at risk of poor glycemic outcomes.

In conclusion, very low state anxiety levels among caregivers of youth with T1D may serve as a clinical signal that these youth are at risk for poor glycemic control. The relationship between caregiver anxiety and glycemic outcomes appears to be complex and may be influenced by factors such as caregiver engagement and parenting style, which warrant further study. Additionally, anxiety symptoms among youth with T1D may be highly prevalent in certain populations. Further research is needed to determine whether and which type (state or trait) of routine anxiety screening for caregiver and youth, coupled with appropriate psychological support, may improve glycemic control and mental health among youth with T1D.

## Data Availability

The raw data supporting the conclusions of this article are available in de-identified form. Inquiries regarding these raw data can be directed to the corresponding author.

## Glossary

Caregiver: Refers to the family member responsible for >50% of the child's diabetes care
Dyad: Refers to a pair, in the case of this study, of the child and their primary caregiver
