# Supplementary material for: Low caregiver state anxiety is associated with worse glycemic control in youth with type 1 diabetes mellitus: a cross-sectional study
Source: Front Pediatr. 2026 Jun 24;14:1806430. doi: 10.3389/fped.2026.1806430 (PMC13341535; doi:10.3389/fped.2026.1806430)
Supplement: Supplementary file 1 [file Datasheet1.pdf]

Supplemental Material 1. Demographic and Clinical Data Survey

Demographic information	
Patient age (in years and months, ex. 12,6 = 12 years 6 months)	________________________
Patient gender	◯ Female<br>◯ Male<br>◯ Other
Caregiver age (in years)	________________________
Caregiver gender	◯ female<br>◯ male<br>◯ other
Patient country of residence	________________________
Patient's city governorate (قضاء) of residence	________________________
Family income level (per month income, if family willing to share)	◯ less than $450<br>◯ between $450 and $800<br>◯ between $800 and $1500<br>◯ between $1501 and $4000<br>◯ greater than $4000
How many people live in the patient's household?	________________________
How many bedrooms are in the household?	________________________
Parents' relationship	◯ married<br>◯ divorced<br>◯ widowed father<br>◯ widowed mother<br>◯ unmarried and living together<br>◯ unmarried and not living together
Family structure	◯ single family<br>◯ multi-family household (2 or more families)<br>◯ multigenerational household (3 or more generations, ex. grandparents, parents, children)
Does the patient live between more than one household?	◯ Yes<br>◯ No
If yes, how many households?	________________________
Caregiver related information	
Primary caregiver's relationship to patient	◯ mother<br>◯ father<br>◯ grandmother<br>◯ grandfather<br>◯ sibling<br>◯ aunt (either maternal or paternal)<br>◯ uncle (either maternal or paternal)<br>◯ other
if other, describe primary caregiver's relationship to patient	________________________

Does the caregiver have diabetes?	◯ No<br>◯ Yes- T1DM<br>◯ Yes- insulin dependent T2DM<br>◯ Yes- non-insulin dependent T2DM
Highest education level of primary caregiver	◯ no school<br>◯ primary school<br>◯ secondary school<br>◯ university degree<br>◯ technical degree
Is primary caregiver employed?	◯ Yes<br>◯ No
Number of other caregivers managing diabetes (ex. 1, 2, 3...)	________________________________
How much help does the primary caregiver receive from other family members?	◯ none<br>◯ rare<br>◯ some<br>◯ a lot<br>◯ shares (splits) 50% of the care work with someone else
Clinical information to illicit from caregiver	
Duration of diabetes (in years and months, ex. 3,4 = 3 years 4 months)	________________________________
Insulin regimen	◯ Pump<br>◯ basal-bolus insulin injections<br>◯ combination of short and intermediate acting insulin injections<br>◯ other
describe insulin regimen if other	________________________________
Co-morbid conditions other than diabetes (according to caregiver)	☐ none<br>☐ thyroid disease<br>☐ celiac disease<br>☐ seizure disorder<br>☐ other
If other, list co-morbid conditions	________________________________

Frequency of hypoglycemia (blood glucose < 70) in the last month

	none	very few times (< 3x/mo = 0-1x/wk)	a few times (4-8x/mo = 1-2x/wk)	frequently (9-15x/mo = 3-4x/wk)	almost daily	unable to assess	n/a
Youth recall	○	○	○	○	○	○	○
Caregiver recall	○	○	○	○	○	○	○
Frequency of hypoglycemia according to conventional glucometer data (if available, measured as "percent of time in hypoglycemia") in the last month	○	○	○	○	○	○	○
Frequency of hypoglycemia according to conventional glucometer data (if available, measured as "percent of time in hypoglycemia") in the first two weeks of the last month	○	○	○	○	○	○	○
Frequency of hypoglycemia according to conventional glucometer data (if available, measured as "percent of time in hypoglycemia") in the last two weeks of the last month	○	○	○	○	○	○	○
Frequency of hypoglycemia according to CGM/Freestyle data (if available, measured as "percent of time in hypoglycemia") in the last month	○	○	○	○	○	○	○
Frequency of hypoglycemia according to CGM/Freestyle data (if available, measured as "percent of time in hypoglycemia") in the first two weeks of the last month	○	○	○	○	○	○	○
Frequency of hypoglycemia according to CGM/Freestyle data (if available, measured as "percent of time in hypoglycemia") in the last two weeks of the last month	○	○	○	○	○	○	○

Did your child lose consciousness or have a seizure because of a low blood sugar

	yes	no
ever in his/her life?	○	○
in the last 3 months	○	○

Has your child ever been in DKA?

- ○ No
- ○ Yes-only at diagnosis
- ○ Yes-only once after diagnosis
- ○ Yes-more than once

How often does your child check his/her blood sugar?

	less than a few times a week	a few times a week	once a day	2-3 times a day	4-5 times a day	more than 5 times a day	unknown	n/a
Youth recall	◯	◯	◯	◯	◯	◯	◯	◯
Caregiver recall	◯	◯	◯	◯	◯	◯	◯	◯
Conventional glucometer data (daily average for the previous month)	◯	◯	◯	◯	◯	◯	◯	◯
Conventional glucometer data (in 1st 2 weeks of the last month)	◯	◯	◯	◯	◯	◯	◯	◯
Conventional glucometer data (in last 2 weeks of the last month)	◯	◯	◯	◯	◯	◯	◯	◯
CGM/Freestyle data (daily average for the previous month)	◯	◯	◯	◯	◯	◯	◯	◯
CGM/Freestyle data (in 1st 2 weeks of the last month)	◯	◯	◯	◯	◯	◯	◯	◯
CGM/Freestyle data (in last 2 weeks of the last month)	◯	◯	◯	◯	◯	◯	◯	◯

Who counts carbohydrates?

- ◯ caregiver
- ◯ youth
- ◯ both
- ◯ N/A if not counting carbohydrates

Who gives the youth insulin?

- ◯ caregiver
- ◯ youth
- ◯ both

Who decides how much insulin to give?

- ◯ caregiver
- ◯ youth
- ◯ both
- ◯ N/A (fixed dosing)

Do you ever have trouble getting medications and supplies?

- ◯ Yes
- ◯ No

Is paying for medication and supplies a financial burden?

- ◯ Yes
- ◯ No

**Data to collection from chart review**	
Most recent HbA1c	______________________
Date of most recent HbA1c	______________________
Tanner stage (breast for females, testicles for males)	◯ I<br>◯ II<br>◯ III<br>◯ IV<br>◯ V<br>◯ unable to assess
Height (cm)	______________________
Weight (kg)	______________________
BMI	______________________
Co-morbid conditions other than diabetes (according to chart review)	☐ none<br>☐ thyroid<br>☐ celiac disease<br>☐ seizure disorder<br>☐ other
List other co-morbid conditions	______________________
