# Supplementary material for: Low caregiver state anxiety is associated with worse glycemic control in youth with type 1 diabetes mellitus: a cross-sectional study
Source: Front Pediatr. 2026 Jun 24;14:1806430. doi: 10.3389/fped.2026.1806430 (PMC13341535; doi:10.3389/fped.2026.1806430)
Supplement: Supplementary file 2 [file Table1.pdf]

Supplemental Table 1. Demographics of consenting versus non-consenting eligible youth

	Consented N=200	Refused N=21	p-value
**Age, yrs** mean ±SD [range]	14.1 ± 1.7 [11.0 – 17.0]	14.4 ± 1.7 [11.5 - 16.9]	0.39
**Females , n (%)**	111 (55.5)	10 (47.6)	0.50
**Age 11-13 yrs, n (%)**	59 (29.5)	4 (19.0)	0.45
**Diabetes duration, yrs** mean ±SD [range]	5.8 ± 3.2 [1.0 - 15.3]	5.8 ± 2.2 [1.9 -10.4]	0.98
**HbA1c (%; mmol/mol)** mean ±SD [range]	8.5 ± 1.6 [5.2 - 15.0]; 69.4 ± 17.5 [33.3-140.4]	8.8 ± 1.7 [6.6 - 13.5]; 72.7 ± 18.6 [48.6-124.0]	0.34
