# Supplementary material for: Low caregiver state anxiety is associated with worse glycemic control in youth with type 1 diabetes mellitus: a cross-sectional study
Source: Front Pediatr. 2026 Jun 24;14:1806430. doi: 10.3389/fped.2026.1806430 (PMC13341535; doi:10.3389/fped.2026.1806430)
Supplement: Supplementary file 3 [file Table2.pdf]

Supplemental Table 2. Association between caregiver STAI state and HbA1c as an interval variable

**Caregiver STAI state score**	**All comers (n=200)** **Adjusted mean ± Standard error [Beta ± standard error]**	**Ages 11-13y (n=59)** **Adjusted mean ± Standard error [Beta ± standard error]**	**Ages >13-17y (n=141)** **Adjusted mean ± Standard error [Beta ± standard error]**
**Bottom quartile** (score 20-24)	9.18 ± 0.28 **p-value 0.002** vs middle 50% [0.87 ± 0.28]	8.51 ± 0.38 **p-value 0.21** vs middle 50%	9.21 ± 0.28 **p-value 0.004** vs middle 50% [1.01 ± 0.34]
**Middle 50%** (score 25-42)	8.30 ± 0.21 **reference**	8.04 ± 0.24 **reference**	8.20 ± 0.19 **reference**
**Top quartile** (score 43-72)	8.71 ± 0.26 **p-value 0.13** vs middle 50% [0.40 ± 0.26]	7.82 ± 0.36 **p-value 0.61** vs middle 50%	8.86 ± 0.27 **p-value 0.046** vs middle 50% [0.66 ± 0.33]
**Frequency of hypoglycemia – caregiver:** **1=none** **2=very few <3x/mo** **3=few 4-8x/mo** **4=frequent 9-15/mo** **5=almost daily**	[-0.29 ± 0.09; p=0.002]	[-0.32 ± 0.15; p=0.03]	[-0.25 ± 0.12; p=0.04]
**Living in a single-family home (yes)**	[-0.37 ± 0.37]	N/A	N/A
	Caregiver STAI state p-value 0.007 Overall model p-value 0.0002 R-square 0.11	Caregiver STAI state p-value 0.43 Overall model p-value 0.03 R-square 0.14	Caregiver STAI state p-value 0.008 Overall model p-value 0.002 R-square 0.10

The all youth model is adjusted for frequency of hypoglycemia over the last month as reported by the caregiver and living in a single-family home. Models by age group are adjusted for frequency of hypoglycemia only.
