# Supplementary material for: Low caregiver state anxiety is associated with worse glycemic control in youth with type 1 diabetes mellitus: a cross-sectional study
Source: Front Pediatr. 2026 Jun 24;14:1806430. doi: 10.3389/fped.2026.1806430 (PMC13341535; doi:10.3389/fped.2026.1806430)
Supplement: Supplementary file 4 [file Table3.pdf]

Supplemental Table 3. Association between caregiver STAI state as an interval variable and HbA1c as an interval and categorical variable

	Including linear, squared and cubic STAI state score terms		Including only the linear STAI state score term	
**Caregiver STAI state score**	**HbA1c Interval β (standard error)**	**HbA1c Categorical Odds ratio (95%CI)**	**HbA1c Interval β (standard error)**	**HbA1c Categorical Odds ratio (95%CI)**
**STAI state score linear**	**-0.82 (0.24) p-value 0.001**	**2.46 (1.18, 5.14) p-value 0.024**	**-0.004 (0.010) p-value 0.65**	**1.01 (0.98, 1.04) p-value 0.49**
**STAI state score squared**	**0.02 (0.006) p-value 0.001**	**0.98 (0.96, 0.99) p-value=0.024**	**N/A**	**N/A**
**STAI state score cubed**	**-0.0001 (0.00005) p-value 0.004**	**1.00 (1.00, 1.00) p-value 0.036**	**N/A**	**N/A**
**Frequency of hypoglycemia – caregiver:** 1=none 2=very few <3x/mo 3=few 4-8x/mo 4=frequent 9-15/mo 5=almost daily	**-0.50 (0.37) p-value 0.17**	**3.67 (0.98, 13.8) p-value=0.054**	**-0.35 (0.38) p-value 0.36**	**2.96 (0.82, 10.7) p-value 0.10**
**Living in a single-family home (yes)**	**-0.29 (0.09) p-value 0.002**	**1.45 (1.10, 1.92) p-value=0.009**	**-0.32 (0.09) p-value 0.001**	**1.47 (1.12, 1.92) p-value 0.006**
	Overall model p-value 0.0002 R-square 0.12	Overall model p-value 0.002 R-square 0.13	Overall model p-value 0.008 R-square 0.06	Overall model p-value 0.01 R-square 0.08

Models are adjusted for frequency of hypoglycemia over the last month as reported by the caregiver and living in a single-family home.
A cubic term was included in the model due to the original hypothesis that both low (bottom quartile) and high anxiety in the caregiver (top quartile) would be associated with higher HbA1c (> 7.5%) in the youth.
