# Supplementary material for: Low caregiver state anxiety is associated with worse glycemic control in youth with type 1 diabetes mellitus: a cross-sectional study
Source: Front Pediatr. 2026 Jun 24;14:1806430. doi: 10.3389/fped.2026.1806430 (PMC13341535; doi:10.3389/fped.2026.1806430)
Supplement: Supplementary file 5 [file Supplementaryfile1.docx]

**Supplemental Methods**

**The alpha coefficients for the anxiety measures used in the study:**

1. **The State-Trait Anxiety Inventory:**

The State-Trait Anxiety Inventory (STAI) was adapted and validated by Abdullatif (15) using a sample of bilingual university students at the American University of Beirut. The study assessed both the original American STAI and a newly developed Arabic adaptation.

American STAI (administered to the Lebanese sample): For Lebanese students responding to the English version, the alpha coefficients were strong across genders

- S-Anxiety (State): α=0.93 for both females and males
- T-Anxiety (Trait): α=0.93 for both females and males

Arabic STAI (Final Adapted Version): The 40-item Arabic adaptation showed even further improvement in internal consistency during validation

The alpha coefficients for its subscales were:

- S-Anxiety Absent: α=0.926
- S-Anxiety Present: α=0.911
- T-Anxiety Absent: α=0.910
- T-Anxiety Present: α=0.905

1. **The Screen for Child Anxiety Related Emotional Disorders (SCARED)**

The Arabic version of SCARED (parent and child) was validated by Hariz et al (17) in a sample of children and adolescents in Lebanon, and demonstrated satisfactory psychometric properties:

The alpha coefficients for the total scales were:

- SCARED-P (Parent version): α=0.92
- SCARED-C (Child version): α=0.91

The internal consistency for the individual subscales (such as Panic Disorder, Generalized Anxiety, and Social Anxiety) ranged between 0.65 and 0.89.
